# Influence of occlusal plane inclination and mandibular deviation on esthetics

**DOI:** 10.1590/2177-6709.20.5.050-057.oar

**Published:** 2015

**Authors:** Cristiane Cherobini Dalla Corte, Bruno Lopes da Silveira, Mariana Marquezan

**Affiliations:** 1 Specialist in Orthodontics, Centro Universitário Franciscano (UNIFRA), Santa Maria, Rio Grande do Sul, Brazil; 2 Professor, Universidade Federal de Santa Maria (UFSM), Department of Restorative Dentistry, Santa Maria, Rio Grande do Sul, Brazil; 3 Postdoc resident, Universidade Federal do Rio de Janeiro (UFRJ), Department of Pediatric Dentistry and Orthodontics, Rio de Janeiro, Rio de Janeiro, Brazil. Dentist, Universidade Federal de Santa Maria (UFSM), Department of Restorative Dentistry, Santa Maria, Rio Grande do Sul, Brazil

**Keywords:** Smiling, Face, Esthetics, Facial asymmetry, Photography

## Abstract

**Objective:**

The aim of this study was to assess the degree of perception of occlusal plane inclination and mandibular deviation in facial esthetics, assessed by laypeople, dentists and orthodontists.

**Methods:**

A woman with 5.88° of inclination and 5.54 mm of mandibular deviation was selected and, based on her original photograph, four new images were created correcting the deviations and creating more symmetric faces and smiles. Examiners assessed the images by means of a questionnaire. Their opinions were compared by qualitative and quantitative analyses.

**Results:**

A total of 45 laypeople, 27 dentists and 31 orthodontists filled out the questionnaires. All groups were able to perceive the asymmetry; however, orthodontists were more sensitive, identifying asymmetries as from 4.32° of occlusal plane inclination and 4.155 mm of mandibular deviation (*p*< 0.05). The other categories of evaluators identified asymmetries and assigned significantly lower grades, starting from 5.88° of occlusal plane inclination and 5.54 mm of mandibular deviation (*p*< 0.05).

**Conclusion:**

Occlusal plane inclination and mandibular deviation were perceived by all groups, but orthodontists presented higher perception of deviations.

## Abstract (pt)

**Objetivo:**

o objetivo do presente estudo foi avaliar o grau de percepção, de leigos, dentistas e ortodontistas, quanto à influência das inclinações do plano oclusal e dos desvios mandibulares na estética facial.

**Métodos:**

uma mulher com 5,88^o^ de desvio do plano oclusal e 5,54mm de desvio mandibular foi selecionada. A partir de sua fotografia original, quatro novas imagens foram criadas, corrigindo os desvios e criando rostos e sorrisos mais simétricos. Os examinadores avaliaram as imagens por meio de um questionário, sendo realizadas análises qualitativas e quantitativas.

**Resultados:**

45 leigos, 27 dentistas e 31 ortodontistas preencheram os questionários. Todos os grupos foram capazes de perceber a assimetria, no entanto, os ortodontistas foram mais sensíveis, sendo capazes de perceber a assimetria de 4,32^o^ de inclinação do plano oclusal e 4,155mm de desvio mandibular (*p* > 0,05). Os demais avaliadores perceberam a assimetria apenas a partir de 5,88^o^ de desvio do plano oclusal e 5,54mm de desvio mandibular (*p* > 0,05).

**Conclusão:**

inclinações do plano oclusal e desvios mandibulares foram percebidos por todos os grupos, mas os ortodontistas apresentaram maior sensibilidade aos desvios.

## INTRODUCTION

Perfect facial symmetry is a theoretical concept. There is no perfectly symmetrical human face, even the most beautiful face exhibits some degree of asymmetry.^1,2^ Asymmetry in craniofacial areas can be recognized as differences in size or relationship between the two sides of the face. This may be the result of discrepancies either in shape of individual bones, or a malposition of one or more bones in the craniofacial complex.^3^ From the point of view of esthetics, it is challenging to establish the threshold level of mild facial asymmetry. It is difficult to find a cutoff point that distinguishes a pleasing asymmetrical face, an acceptable asymmetrical face and an asymmetrical face that requires intervention. Despite the subjectivity of beauty, it becomes necessary to acknowledge and study facial esthetics, bearing in mind the concept of normality which serves as a guide during orthodontic treatment planning.^4^

Craniomandibular structural asymmetry can be congenital or hereditary, or can be acquired as a result of trauma or infection. During growth, quantitative and qualitative alterations of functional loads applied to the bones might modify their developmental pattern and lead to asymmetry.^5^Facial asymmetry may be present in the upper, middle and lower thirds of the face. The majority of asymmetries are usually concentrated in the lower third of the face due to being involved in the masticatory structures^6^^,^ 7 ^,^ 8 and subject to masticatory and occlusal problems.^9^

Many patients with facial asymmetry present occlusal plane inclination caused by unilaterally extruded maxillary molars or asymmetrical mandibular vertical development.^10^ Because the occlusal plane is an important element in the position and adaptation of the mandible,^11^ inclination is usually associated with mandibular deviation and vice-versa.^12,13^ The degree of inclination of the maxilla is proportional to the degree of mandibular deviation in both hard and soft tissues.^13^ The prevalence of inclination is about 41%, but many cases are not perceptible due to being of minor severity.^14^

Facial asymmetries in soft tissues influence patient's expectations regarding orthodontic treatment.^8^ In order to prevent disagreements between patient's and orthodontist's treatment objectives, the normal range of facial asymmetry needs to be determined in a given population.^1^ Therefore, the aim of this research was to assess the influence of occlusal plane inclination and consequent mandibular deviation on esthetics in the opinion of laypeople, dentists and orthodontists.

## METHODS

This study was characterized as an observational, descriptive, transverse study with quantitative and qualitative analysis of data. It was submitted to and approved by the Human Research Ethics Committee of Centro Universitário Franciscano (UNIFRA) (CAAE: 11097113.5.0000.5306, #265.831, issued on 21^st^ of May, 2013). A model with an esthetically pleasant face, but with severe occlusal plane inclination (5.88°) and mandibular deviation (5.54 mm), both of which led her to seek orthodontic treatment, was selected for the study.

**Figure 1:**
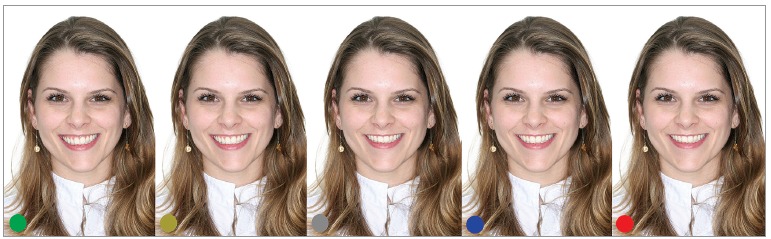
- Original smile (green label), with 5.88° of inclination and 5.54 mm of mandibular deviation, and their corrections (1.47° and 1.385 mm in each photo).

Based on patient's original photograph, a professional designer created four smiles by means of Adobe Photoshop CS5 (Adobe Systems, San Jose, California) software. Firstly, the pupillary plane was traced and positioned parallel to the ground. A new line was traced starting from the center of the left pupil up to the tip of the maxillary left canine cusp.^23^ The distance between these points was transferred to the right side, and, thus, it was possible to trace the ideal occlusal plane. The difference between the angle formed by the patient's real inclination and the digital manipulation at an angle equal to zero resulted in 5.88°. The four manipulated smiles had occlusal plane inclination progressively corrected by 1.47° in each photo, until the smile became symmetrical. As the occlusal plane was being altered, mandibular deviation was also manipulated until it was completely corrected (1.385 mm for each photograph, totaling 5.54 mm), rendering the face more symmetrical and making the facial midline match with the center of the mentum (Fig 1). It is emphasized that the model agreed with the use of her image in the research by signing a term of authorization for image use.

The five images were identified with colored labels (Fig 1) and randomly disposed in a photograph album. There was only one photograph on each page of the album, so that the images were not compared side by side. The evaluators were not allowed to return to the previous photo or move on without attributing a score to the photograph. No set time was established for the evaluations. The albums were made available to three categories of evaluators: laypeople, dentists and orthodontists; together with a questionnaire in which the evaluators could express their esthetic preference by attributing scores from 0 to 10 (zero to ten) to each image. In the questionnaire, the evaluators were also asked whether they perceived anything that called their attention in each one of the photographs, so as to justify the score attributed to them.

A total of 150 questionnaires were distributed in Dental Schools and Dental Clinics of Santa Maria (Rio Grande do Sul, Brazil). These are the places where dentists and orthodontists work, and laypeople can also be found (employees, patients' relatives and friends).

Quantitative data were tabulated in SPSS Statistics version 20 for statistical analysis. The scores attributed to each smile in the categories of evaluators were compared by ANOVA/Tukey tests.

## RESULTS

Of the 150 questionnaires, 103 were returned duly filled out: 45 by laypeople, 27 by dentists and 31 by orthodontists. All groups were able to perceive asymmetry; however, orthodontists were more sensitive, identifying asymmetries as from 4.32° of inclination and 4.155 mm of mandibular deviation (*p* ≤ 0.05). The other categories of evaluators identified asymmetries and assigned significantly lower grades, starting from 5.88° of inclination and 5.54 mm of mandibular deviation (*p* ≤ 0.05). All three groups of evaluators considered the original photograph (green) as the least attractive one, followed by gold and silver photographs. Laypeople and orthodontists considered the most symmetrical face and smile (red photograph) to be the most attractive. Dentists, on the other hand, preferred the blue photograph. The results of the qualitative and quantitative analyses (means, standard deviation and results of ANOVA/Tukey tests) as regards the esthetic preference of laypeople are shown in Table 1, whereas the preference of dentists is shown in Table 2 and orthodontists' preference is shown in Table 3.

**Table 1:** - Perception of laypeople.

**Preferably sequence**	**Smile**	**Qualitative analysis**	**Quantitative analysis mean (SD)**	ANOVA/ Tukey*
1^st^	Red	» White teeth (3 people) » Perfect teeth, aligned (3 people) » Thin upper lip (3 people) » Gingival exposure (2 people) » Harmony (2 people) » Deviated chin » Narrow mouth » Narrow smile » Aligned chin » Different earrings » Happiness	8.68 (1.39)	a
2^nd^	Blue	» Good gingival exposure (5 people) » White teeth (4 people) » Bent smile (3 people) » Beautiful teeth, perfect (3 people) » Problem in the height of the teeth » The right side of the patient is higher » Thin upper lip » Problems in the teeth, gingiva and lip » Great alignment » The smile expresses pleasure, happiness, sympathy	8.60 (1.13)	a
3^rd^	Silver	» Asymmetry (6 people) » Yellow teeth (2 people) » Lighten teeth (2) » Perfect teeth (2) » Deviated chin » Difference in posterior teeth » Thin upper lip » Beautiful smile » Mouth and face in harmony » Happiness, sympathy	8.31 (1.25)	a,b
4^th^	Gold	» Asymmetric smile (13 people) » White teeth (3 people) » Symmetric teeth (3 people) » Happy person (2 people) » Beautiful smile (2 people) » Asymmetric face » Deviated chin » One side of the mouth is more open » Aligned teeth » Happiness, sympathy in the look and smile	8.04 (1.52)	a,b
5^th^	Green	» Asymmetric smile (20 people) » One side of the mouth is more open » Bent upper lip » Deviated chin » Posterior teeth are different » Lighten teeth, perfect » Smile revels teeth well » Happiness, sympathy	7.75 (1.54)	b

* ANOVA showed statistical difference among groups (*p* = 0.016). Different letters indicate statistical difference for post hoc Tukey.

**Table 2:** - Perception of dentists.

**Preferably sequence**	**Smile**	**Qualitative analysis**	**Quantitative analysis mean (SD)**	ANOVA/ Tukey*
1^st^	Blue	» Lower midline deviation (7 people) » Inclination (4 people) » Smile line (2 people) » Chin deviation (2 people) » Increased buccal corridor » Small left eye » Bent nose » Thin upper lip	8.42 (1.11)	a
2^nd^	Red	» Lower midline deviation (8 people) » Low smile line (8 people) » Chin deviation (2 people) » Narrow buccal corridor (2 people) » Thin upper lip » Seems to have many teeth » Tooth 23 bucally positioned	8.13 (1.50)	a
3^rd^	Silver	» Inclination (11 people) » Lower midline deviation (8 people) » Chin deviation (5 people) » Low smile line » Acute zenith on the right side » Buccal corridor » Color of the teeth » Tooth 23 bucally positioned	7.85 (1.26)	a,b
4^th^	Gold	» Inclination (20 people) » Lower midline deviation (11 people) » Chin deviation » Small left eye » Increased buccal corridor	7.50 (1.13)	a,b
5^th^	Green	» Inclination (23 people) » Lower midline deviation (7 people) » Chin deviation (2 people) » Buccal corridor (2 people) » Tooth 23 bucally positioned	6.87 (1.51)	b

* ANOVA showed statistical difference among groups (*p* = 0.000). Different letters indicate statistical difference for post hoc Tukey.

**Table 3:** - Perception of orthodontists.

**Preferably sequence**	**Smile**	**Qualitative analysis**	**Quantitative analysis mean (SD)**	ANOVA/ Tukey*
1^st^	Red	» Lower midline deviation (15 people) » Chin deviation (6 people) » Little gingival exposure (4 people) » Artificial smile (3 people) » Larger buccal corridor on left side » Greater exposure of left teeth » Decreased vertical dimension	8.48 (1.37)	a
2^nd^	Blue	» Lower midline deviation (17 people) » Mandibular asymmetry (8 people) » Inclination (3 people) » Thin upper lip (2 people) » Nose (2 people) » Little exposure of lower incisors » Mild crowding of tooth #21 » Low smile line	8.15 (0.74)	a,b
3^rd^	Silver	» Inclination (15 people) » Mandibular asymmetry (11 people) » Lower midline deviation (11 people) » Upper lip (3 people) » Buccal corridor » Nose	7.58 (1.10)	a,b
4^th^	Gold	» Inclination (22 people) » Mandibular asymmetry (10 people) » Lower midline deviation (9 people) » Buccal corridor » Little exposure of lower incisors » Nose	7.25 (1.05)	b
5^th^	Green	» Inclination (24 people) » Mandibular asymmetry (11 people) » Lower midline deviation (8 people) » Buccal corridor (2 people) » Upper central incisor inclined to the left » Nose	6.20 (1.58)	c

* ANOVA showed statistical difference among groups (*p* = 0.000). Different letters indicate statistical difference for post hoc Tukey.

## DISCUSSION

Although no face is perfectly symmetrical, a face is only considered asymmetrical when there is perceptible disharmony between homologous parts. It is known that a certain degree of asymmetry is beautiful, but the border line between normal asymmetry and asymmetry that requires treatment is subjective^15^ and varies among professionals and laypeople.^16^ In order to assess the threshold of esthetic tolerance for occlusal plane inclination and mandibular lateral deviation, the photographs of a model with an asymmetrical face were gradually edited until these parameters became symmetrical. In the qualitative analysis, it was perceived that the largest number of evaluators detected the asymmetry of the smile (inclination) rather than that of the face itself (mandibular shift). In the three categories, a lower number of evaluators reported deviation of the mandible or chin. According to a previous study, frontal photographs of the face allow facial symmetry, and numerous other factors such as eyes, size and shape of the face, to be assessed. These aspects can divert attention from potential skeletal-facial changes.^17^

Laypeople, dentists and orthodontists were capable of perceiving occlusal plane inclination associated with mandibular lateral deviation. The original photograph (green), with an inclination of 5.88° and 5.54 mm of lateral deviation of the mandible, was the one that received the lowest score in the three categories, followed by photographs with gold (4.32° and 4.155 mm of deviation) and silver (2.88° and 2.77 mm of deviation) labels. The photographs with blue and red labels received the highest scores, being red (symmetrical) preferred by laypeople and orthodontists. Nevertheless, the photograph with a blue label was preferred by dentists who probably did not identify the small degree of inclination (1.47°) and small mandible deviation (1.385 mm) and perceived the more harmonious smile line (Table 2). It has been shown that a smile with the upper lip resting on the gingival margin of maxillary incisors (as found in the blue photograph) is considered the most esthetic for a female subject.^18^

Orthodontists were more sensitive to perceiving inclination and chin deviation. When verifying the scores attributed to the photographs, orthodontists assigned significantly lower grades (*p* ≤ 0.05) to the smile in the gold label photograph (*p* ≤ 0.05) (Table 3). This means they identified asymmetries as from 4.32° of inclination and 4.155 mm of mandibular deviation. Unlikewise, dentists and laypeople assigned significantly lower grades (*p* ≤ 0.05) only to green label photographs(Tables 1 and 2). This means that they identified asymmetries as from 5.88° of inclination and 5.54 mm of mandibular deviation. Previous studies have shown lower cutoff values for inclination and mandibular deviation. Padwa et al^19^ considered an inclination of 4° as the threshold for recognition by laypeople and trained evaluators. For mandibular deviation, Da Silva et al^20^ found that orthodontists and laypeople only perceived shifts greater than or equal to 4 mm when analyzing a woman's photographs (rest position). When examining a man's photographs, orthodontists perceived shifts greater than or equal to 4 mm, but laypeople did not perceive any changes (up to 6 mm). On the other hand, in a classification proposed by Kim et al,^13^used to assess facial asymmetry in diagnosis for orthognathic surgeries, mandibular and occlusal plane deviations greater than 2 mm were considered as asymmetries.

Although some laypeople perceived inclination and mandibular deviation, these deviations were mentioned by a larger number of orthodontists. While dentists and orthodontists analyzed the smile more carefully, detailing problems such as buccal corridor and midline deviation, the attention of laypeople did not focus so much on the oral region, as they reported details of the face other than the teeth, such as earrings and eyes, in addition to subjective characteristics such as sympathy and happiness. According to Jackson et al,^21^orthodontists have a clear advantage in assessing facial symmetry when compared with laypeople, and an advantage over general clinical dentists in the most difficult cases.

The more symmetrical the smile, the more details, such as color and anatomy of teeth, were perceived and described by the interviewees; however, with increasing inclination and mandibular deviation, the negative influence of these features was perceived and described by the three groups of evaluators.

Although laypeople perceived the asymmetries, the mean scores attributed to the photograph with green label (the most asymmetric one) was still high (7.75). This fact makes one wonder if this amount of asymmetry is clinically significant for patients as far as esthetics is concerned.

Some limitations were detected and need to be addressed by future studies, such as the number of questionnaires. Sample size should be increased to reduce the possibility of type II error (failure to reject a false null hypothesis; in other words, failure to detect difference between photographs). It would also be interesting to use different models, varying the sex, ethnicity, color of the hair, skin, and eyes. It would also be interesting to register more detailed data about the evaluators, such as the time elapsed since their graduation (experience time).

## CONCLUSION

The three groups of evaluators perceived inclination and mandibular deviation; however, orthodontists were those with the greatest perception.
